# Codes, care, and consequence: A viewpoint on CPT valuation in obstetric practice

**DOI:** 10.1002/pmf2.70359

**Published:** 2026-07-06

**Authors:** Brian K. Iriye

**Affiliations:** ^1^ High Risk Pregnancy Center Hera Women's Health Las Vegas Nevada USA

**Keywords:** CPT coding, maternal‐fetal medicine, obstetrics, reimbursement, relative value unit, ultrasound

## Abstract

In 2027, obstetric care in the United States will be billed differently than it has been for nearly half a century. Long‐standing maternity global codes will be retired and replaced by new and revised codes that separately report antepartum, labor management, delivery, and postpartum care. The valuation of these codes, and of the broader obstetric ultrasound family, is determined through the AMA/Specialty Society Relative Value Scale Update Committee (RUC) process and is constrained by statutory budget neutrality within the Medicare Physician Fee Schedule. This viewpoint argues that CPT valuation in obstetrics has consequences at the bedside and is supported by two arguments. First, valuation latency—the interval between a code's last survey and the current state of the work it describes—is itself a clinical problem, and the obstetric ultrasound family, in which expanding guideline content has outpaced formal revaluation, is the clearest current example. Second, the rigor of the specialty‐society survey process is the proximate determinant of whether new and revised codes are valued accurately, and that rigor depends directly on the time and care physicians invest in their responses. The viewpoint is directed at maternal‐fetal medicine and obstetric clinicians, providing background on the CPT valuation process and underscoring that thoughtful participation in specialty‐society RUC surveys is a meaningful contribution to accurate valuation.

## 1 INTRODUCTION

In 2027, obstetric care in the United States will be billed differently than it has been for nearly half a century. The American Medical Association has approved the deletion of several long‐standing maternity Current Procedural Terminology (CPT) codes, including the global codes, and their replacement with new and revised codes that separately report antepartum, labor management, delivery, and postpartum care [1]. Antepartum and postpartum visits will move to per‐encounter evaluation and management reporting, labor management will be reported daily by complexity, and delivery codes will report delivery alone.

The position of this viewpoint is that CPT valuation in obstetrics has implications and consequences at the bedside. Two arguments support that position. First, valuation latency is itself a problem: codes not reviewed for many years reflect a prior era of equipment, staffing, and practice, and the obstetric ultrasound family is the clearest current example. To describe this further, valuation latency is the interval between the last survey of a code's relative value units and the current state of the work it describes. In ultrasound specifically, over an extended time interval, equipment improves or becomes obsolete, image‐acquisition and interpretation standards expand, documentation requirements grow, and the personnel and physical infrastructure required to perform the service evolve. When the interval spans decades, the code's assigned work, time, and practice expense no longer correspond to current effort or resource use, and the gap quietly compounds because budget neutrality provides no mechanism for retrospective correction. Second, the rigor of the survey process determines whether new and revised codes are valued accurately, making the time physicians invest in their responses an important evaluation of clinical input rather than a clerical matter.

## 2 HOW A CPT CODE IS VALUED

The Centers for Medicare & Medicaid Services (CMS) sets payment for physician services through the Resource‐Based Relative Value Scale. Each CPT code has a total relative value unit (RVU) composed of the average physician work (approximately 51%), practice expense (approximately 45%), and professional liability insurance (approximately 4%) [2]. Physician work is intended to capture time, technical skill, physical effort, mental effort, clinical judgment, and the stress associated with patient risk.

When a code is new, substantially revised, or flagged for review, the AMA/Specialty Society Relative Value Scale Update Committee—commonly referred to as the RUC, which has 32 members—is engaged. The RUC is a multispecialty expert panel, supported by specialty society advisors, that develops recommendations to CMS on the resources required to perform physician services, shaping the RVUs assigned to CPT codes. One seat represents Obstetrics and Gynecology and this is held by the American College of Obstetrics and Gynecology (ACOG). Maternal‐Fetal Medicine is not seated separately and currently contributes to obstetric code deliberations only through ACOG's delegation. To build those recommendations to the CMS, the RUC asks the relevant specialty societies to survey their members. Respondents estimate pre‐, intra‐, and post‐service time, compare the service to reference codes of known intensity, and provide their own estimate of the work RVU. The society develops a recommendation and presents it to the RUC, which forwards it to CMS [3]. The survey is open to any practicing physician within the society's membership who performs the code, a deliberate design intended to reflect the full population of physicians who bill it. That openness is a feature with consequences.

Compounding these pressures, CMS operates the Physician Fee Schedule under a statutory budget‐neutrality requirement: under Section 1848 of the Social Security Act as changes to relative value units may not alter projected annual expenditures by more than $20 million per year‐ a threshold unchanged since 1992 [4, 5]. Any increase in the value of one service is therefore offset by a downward adjustment to the conversion factor across all other services, and revaluation rarely stops at a single code, as the RUC's relativity review typically extends to the surrounding code family.

**FIGURE 1:**
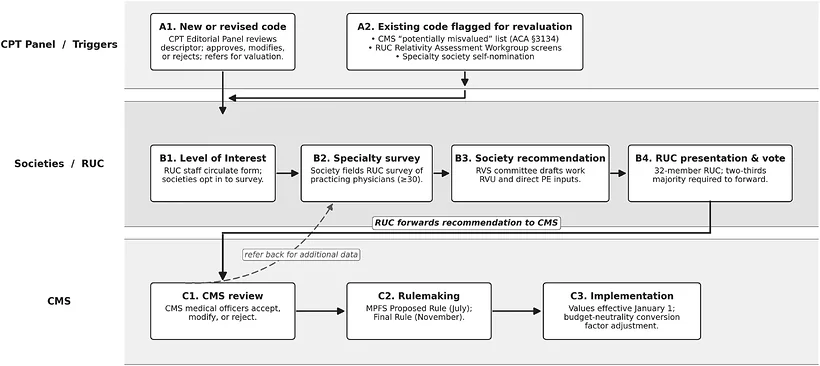
Pathway for valuation and revaluation of a Current Procedural Terminology (CPT) code under the Medicare Physician Fee Schedule (MPFS). ACA, affordable care act; CMS, Centers for Medicare & Medicaid Services; RUC, AMA/Specialty Society Relative Value Scale Update Committee; RVS, relative value scale; RVU, relative value unit. Schematic synthesized from the AMA RUC process descriptions [3, 6].

Codes enter the revaluation queue through three channels: CMS designation as potentially misvalued, identification by the RUC's Relativity Assessment Workgroup through objective screens such as volume growth, site‐of‐service anomalies, and time since last survey, and nomination by specialty societies themselves (see Figure 1).

## 3 CPT 76811: EXPANDING CONTENT, STATIC VALUATION

The detailed fetal anatomic ultrasound examination, CPT 76811, was introduced in the 2003 edition of the CPT manual as a referral‐level study distinct from the standard second‐trimester examination. Its initial valuation was developed through a robust specialty‐society survey process, and the resulting RVU was widely viewed as commensurate with the work of a detailed referral examination at that time. Uptake was correspondingly broad, and 76811 became established as the principal code for the detailed anatomic survey in higher‐risk pregnancies.

In the years that followed, the content of the examination did not remain static. The 2014 Society for Maternal Fetal Medicine (SMFM) consensus document and the 2019 American Institue of Ultrasound in Medicine (AIUM) practice parameter together codified a substantially expanded catalog of detailed anatomic elements—across cardiac, central nervous system, facial, placental, and extremity systems, among others—that may be required of a 76811 examination depending on clinical indication [7, 8]. The 2019 parameter does not enumerate a universally required list, but the responsible physician must possess the training, real‐time pattern recognition, and reporting depth to execute any catalog element on demand, with additional elements when warranted by findings. Because 76811 includes all components of 76805, the expansion of 76805's required components through successive AIUM revisions through 2024 [9] has further raised the mandatory floor of 76811. The structural problem is not that 76811 was undervalued at inception; it is that the content and expertise the code now represents have expanded through successive guideline revisions while the valuation has not been formally revisited—the kind of mismatch the RUC re‐review process exists to correct. The patient population has also shifted: maternal obesity in the United States has roughly doubled since the early 2000s, and obesity degrades acoustic windows, lengthens acquisition time, and increases the effort required to complete each examination—all elements RVU work is intended to capture.

## 4 THE TECHNICAL COMPONENT

For diagnostic imaging, the practice expense component is frequently the larger share of the total RVU and is most directly tied to what the obstetric ultrasound suite must purchase, employ, and maintain. The direct survey inputs of cost reviewed in the RUC process include clinical staff time, medical supplies, and medical equipment. For the obstetric ultrasound family, those variables are concrete: sonographer labor; the ultrasound system, transducers, and workstation with their lease, service, and replacement costs; and the consumables and staff time required for high‐level disinfection of endocavitary transducers between patients. The sonographer market in 2026 is tighter and more expensive than the market against which 76811 was originally valued, and equipment costs have not remained static. When a code in the family is reviewed, these inputs must be reconstructed against contemporary practice and expanding documentation requirements rather than the assumptions embedded in the original valuation.

The expanded content of the obstetric ultrasound examinations has its own equipment requisites. Reliable acquisition and documentation of the components now expected of these examinations necessitates contemporary high‐resolution transducers, color and pulsed Doppler capability, and image‐processing performance that exceeds what was sufficient at inception [8]. Current parameters also recommend video clips for selected views and archiving of images with the final report, which require additional equipment, storage, and information‐system infrastructure not contemplated by the original valuations. Equipment alone, however, is not sufficient: the practical yield of a contemporary ultrasound system depends on the operator who drives it. Sonographer and physician expertise has had to keep pace with the equipment, and the cost of recruiting and retaining personnel capable of performing these examinations to the current standard is part of the practice expense the code is intended to cover.

## 5 CODES DRIFT WHEN THEY ARE NOT REVIEWED

It is sometimes assumed that once a CPT code is valued, the value persists indefinitely. The opposite is true. The Medicare Physician Fee Schedule is updated annually, and codes are routinely identified for re‐review through the AMA Relativity Assessment Workgroup, the CMS potentially misvalued code initiative, and changes in utilization, technology, or practice patterns. Codes not reviewed in many years therefore carry valuations grounded in an earlier era. CPT 76805 illustrates the pattern. The AIUM practice parameter for standard diagnostic obstetric ultrasound has been revised multiple times, most recently in 2024, and each revision has expanded the required content of the examination. The cardiac examination has progressively tightened: views of the right and left ventricular outflow tracts moved from a conditional element to be attempted when technically feasible to expected components of a complete examination, and the three‐vessel and three‐vessel‐trachea views and fetal cardiac axis assessment have been added as expected elements. The placental cord insertion site, beyond the originally specified fetal abdominal insertion, is now expected when technically possible, with color and pulsed Doppler interrogation when a velamentous insertion or vasa previa is suspected. Cervical evaluation and measurement, previously qualified as when appropriate, is now unconditional. Extremity documentation has shifted from the presence or absence of legs and arms to discrete, labeled views of each of the four extremities, hands, and feet. Two‐dimensional video clips of selected views have moved from absent to an accepted and at times expected method of documentation across the examination with archiving of images and the final report now an expected component of the examination record [9]. Several obstetric ultrasound codes—76811 and its add‐on 76812, the standard examinations 76805 and 76810, and the limited and follow‐up examinations 76815 and 76816—have not undergone formal re‐survey and revaluation in many years, despite substantial changes in equipment resolution, image‐acquisition standards, accreditation expectations, and required examination components.

## 6 THE 2027 MATERNITY RESTRUCTURING

The 2027 maternity restructuring will require the valuation of several new codes (59080–59083, 59431–59434, 59502–59504, and 59623) and revaluation of revised codes (59051, 59300, 59412, 59414, 59898, and 59899) [1]. The changes are anticipated to be approximately budget neutral within the obstetric family. How labor management complexity is weighted, how the antepartum E/M structure compares to the previous global, and how delivery‐only codes are valued relative to historical bundled rates will be shaped by the survey responses already submitted.

## 7 WHY SURVEY RIGOR IS NOT A SMALL PROCEDURAL DETAIL

A common assumption is that a single survey response is unlikely to matter. The mathematics of the RUC process suggests otherwise. Specialty society surveys often close with response counts on the order of a few dozen to a few hundred practicing physicians, and the recommendation that reaches the RUC is built around the median, the 25th percentile, and the distribution of those responses [6]. A small number of additional responses that accurately reflect a more time‐intensive pattern of practice can move the median appreciably; a small number of rapid, underestimated responses can shift it in the opposite direction.

Accurate reconstruction means accounting for the full arc of work attributable to the procedure itself for the typical patient: review of the indication and prior records before the patient arrives; the focused history; the procedure or encounter itself; structured documentation of findings; communication of results to the patient and, when relevant, the referring provider; and review and signature of the final report. Work that extends beyond the procedure, such as extended counseling for abnormal findings, complex care planning, and management of a new diagnosis, is properly captured through evaluation and management codes rather than the procedure's work RVU. A response that captures only intra‐service time will systematically understate the procedural work; one that imports work belonging to a separately reportable service will overstate it.

## 8 WHY ACCURATE VALUATION MATTERS AT THE BEDSIDE

The clinical consequences of valuation latency are not abstract. They are present in three patterns. The first is equipment aging: when reimbursement for a high‐complexity ultrasound service has not been recalibrated against the cost of contemporary systems, practices defer replacement and software upgrades, and the detection of a subtle finding on a fetal anatomic survey is, in part, a function of the resolution of the system on which it is performed. The second is workforce attrition: sonographer compensation, physician compensation, and the operational margin available to the supervising clinician are downstream of code value, and when that value lags the cost of service, practices respond first by reducing margin and ultimately by losing experienced personnel.

The third is volume. When reimbursement does not keep pace with cost, the most direct way to maintain revenue is to perform more studies per unit time, and outpatient imaging schedules are unforgiving: more studies per session mean less time per study. Valuation latency, by compressing the time available for each examination, exerts a quiet but cumulative downward pressure on diagnostic yield.

## 9 THE POSITION

CPT valuation in obstetrics has consequences for patients, and the obstetric community should treat it accordingly. Valuation latency is itself a clinical problem, and the obstetric ultrasound family is the clearest current example. The rigor of the RUC survey process is the proximate determinant of whether new and revised codes are valued accurately, and that rigor depends on the time and care physicians invest in their responses. Physicians who receive a survey invitation for a code in which they have direct experience are encouraged to set aside the time to complete it carefully, reconstruct the full pre‐, intra‐, and post‐service workflow, and compare the service honestly with the listed reference codes. The survey is brief. Its consequences and long‐ranging effects are not.

## AUTHOR CONTRIBUTIONS

**Brian K. Iriye**: Conceptualization; writing—original draft; writing—review and editing.

## CONFLICT OF INTEREST STATEMENT

The author owns equity in Hera Women's Health (healthcare organization) and serves as President and Chief Medical Officer.

## FUNDING INFORMATION

The author received no specific funding for this work.

## Data Availability

Data sharing not applicable to this article as no datasets were generated or analyzed during the current study.
